# Supplementary material for: Surgical and Oncological Outcomes of Salvage Hepatectomy for Locally Recurrent Hepatocellular Carcinoma after Locoregional Therapy: A Single-Institution Experience
Source: Cancers (Basel). 2023 Apr 16;15(8):2320. doi: 10.3390/cancers15082320 (PMC10137253; doi:10.3390/cancers15082320)
Supplement: Supplementary file 1 [file cancers-15-02320-s001.zip › cancers-2258263-supplementary.pdf]

## Supplementary Materials

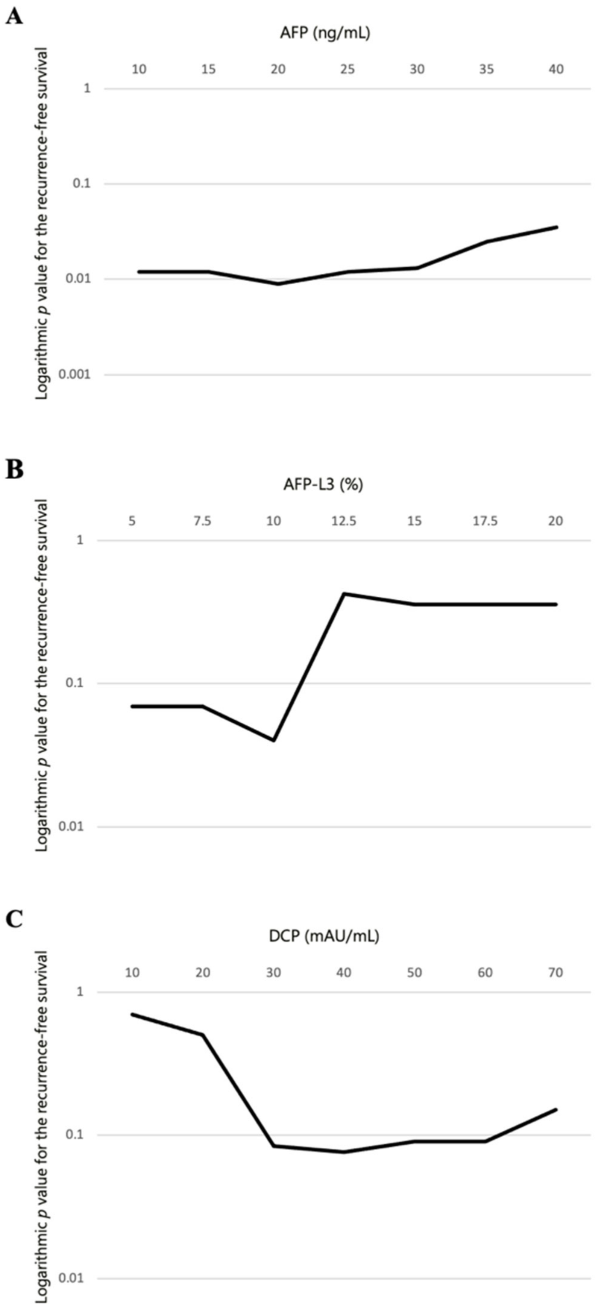


**Figure S1.** Optimal cut-off values of tumor markers based on the prognostic differences in the recurrence-free survival of the patients; 20 ng/mL for AFP ($P$ = 0.009) (A), 10% for AFP-L3 ($P$ = 0.04) (B), and 40 mAU/mL for DCP ($P$ = 0.076) (C).
